# Family size preference and factors affecting the fertility rate in Hyogo, Japan

**DOI:** 10.1186/1742-4755-10-6

**Published:** 2013-01-30

**Authors:** Yasuyo Matsumoto, Shingo Yamabe

**Affiliations:** 1 Department of International Medical Cooperation Japan, National Center for Global Health and Medicine, 1-21-1 Toyama, Shinjuku-ku, Tokyo 162-8655, Japan; 2 Yamabe Ladies Clinic, 2-5-1 Sannomiya, Chuo-ku, Kobe, 650-0021, Japan

**Keywords:** Family size, Low fertility, Japan

## Abstract

**Background:**

Japan has consistently shown a low fertility rate, which has been lower than the replacement level since 1974, and represents one of the least fertile countries in the world. This study was designed to determine the family size preference of and its effect on Japanese women.

**Methods:**

We conducted a questionnaire survey among women who visited the obstetrics and gynecology department of 18 hospitals and clinics in the Hyogo Prefecture, Japan, between October 2011 and February 2012. All the women were categorized according to age group and area of residence, and the survey results were statistically analyzed using a *t* test.

**Results:**

A total of 1616 women were included in this study. There was no significant difference between the mean desired and actual marital ages (26.70 and 26.67 years, respectively). The mean desired number of children was 2.55, which was significantly more than the mean actual number of children (1.77) in all generations. The mean desired and actual numbers of children were more in the rural areas (2.73 and 2.09, respectively) than in the urban (2.54 and 1.70, respectively) and semi-urban areas (2.49 and 1.60, respectively). The mean number of family members was significantly greater in the rural areas (3.84) than in the urban (3.25) and semi-urban areas (3.05).

The most important concern among women who had never delivered a baby was childbearing itself, followed by the expenses related to pregnancy and childbearing.

**Conclusions:**

The family size preference of the women in our study was higher than the actual numbers of children. The fertility intentions were low among the younger women but high among those living in rural areas with larger families.

## Background

A woman’s family size preference is strongly related to her social background. A study in China showed that the preference for a small family was associated with younger age, urban residence, and higher level of education [1]. The National Health Statistics Reports in the United States revealed that the fertility intention of men and women differed across races and religions [2,3]. With regard to religion, Catholic women tended to have fewer children than Protestant women; however, fertility intention was high among Mormons and Hispanics, regardless of their religion, and was lowest among Jewish women and those with no religion [2]. The latest reports have shown that white women had fewer number of children and an older mean age at first birth than Hispanic and black women [3]. Another recent report indicated that men and women with low levels of education were likely to have high mean numbers of children [3].

The total fertility rate (TFR) in Japan was 4.54 in 1947, the year of the “first baby boom,” and decreased gradually to 1.98 in 1962. Although the TFR had been increasing from 1971 to 1974, the period of the “second baby boom,” it started decreasing again in 1974, dropping to 2.05, which was lower than the replacement level. It continued decreasing until it reached its lowest value at 1.26 in 2005. Thereafter, the TFR had slightly increased to approximately 1.3–1.4; however, the trend of having fewer children per family has not changed recently [4].

In the case of China, the preference for a small family in younger age groups in the Chinese population can be attributed to the 1-child policy. However, for Japan, the following questions remain to be answered: What has influenced the decrease in the TFR in Japan? Do Japanese women really desire few children?

To determine the family size preference of Japanese women, we conducted a questionnaire survey among women who visited the obstetrics and gynecology department of 18 hospitals and clinics in the Hyogo Prefecture, Japan. Although our place of study did not provide a study population reflective of the total population in the Hyogo Prefecture, it provided an appropriate venue for asking women questions regarding sensitive topics such as family size preference.

In Japan, health services are provided through public and private hospitals/clinics. The cost of personal medical services is the same regardless of whether a public or private facility is used because standard fees for medical services were set by the Ministry of Health, Labour and Welfare. Patients pay a certain amount of the total fee according to their type of insurance coverage (mostly 30% of the total amount) provided by a universal health-care insurance system. Patients can freely choose physicians or facilities according to their convenience and preference.

The Hyogo Prefecture is located in the middle of the main island of Japan and has a total population of 5.5 million. The capital, Kobe (population, 1.5 million), is located in the southern part of the Hyogo Prefecture and is an important commercial center, with many heavy machinery and chemical industries [5]. Meanwhile, in the thinly populated areas extending from the northern to the central parts of the Hyogo Prefecture, where there is constant heavy snowfall, agriculture, logging, and fishery represent the main industries. With an area of 8,396.16 km^2^, the Hyogo Prefecture comprises both densely and thinly populated commercial and industrial areas; hence, we deemed it possible to compare urban and rural residents in this prefecture.

## Methods

This study was conducted at 18 hospitals and clinics (urban, 8; semi-urban, 5; rural, 4; and island, 1) in Japan’s Hyogo Prefecture between October 2011 and February 2012. All the women who visited the obstetrics and gynecology outpatient department in each facility for the first time were asked to complete a questionnaire. After explaining the objectives of the study, a written consent was obtained from each participant. The questionnaire was anonymous and self-administered, and included questions regarding age, marital status, number of family members, desired marital age, actual marital age, desired number of children, gravidity, parity, history of artificial abortion, and anxiety about pregnancy and childbearing (only for nulliparous women). The women could chose which questions they did not want to answer. The doctor in charge at each hospital and clinic collected and sent the questionnaires to the investigators. Ethical approval (No. 934) was obtained from the ethics committee of the National Center for Global Health and Medicine.

All the women were categorized according to age (20–29, 30–39, 40–49, 50–59, and older than 60 years), residential area (urban, semi-urban, rural, and island), and marital status. However, we were not able distinguish between unmarried statuses (never married or divorced/separated), as questions pertaining to this seemed too sensitive for the Japanese culture. In the statistical analysis, we excluded data from residential areas and marital status groups with fewer than 20 women because this number is too small, although it demonstrated a high deviation rate of 80% in terms of unmarried status. Although the island has agriculture and fishery as main industries, we considered it separate from the rural area because it is located in the warm southern part of the Hyogo Prefecture and has become easily accessible (1 h away) from the capital, Kobe, after a bridge was constructed in 1996, greatly improving the public transportation system. The participants were divided according to age and residential area. The data were statistically analyzed using a *t* test.

## Results

A total of 1616 women were included in this study. The mean ± SD age of the participants was 39.96 ± 11.02 years, and the median age was 34 years. The percentage of unmarried women was 23.01%. The desired marital age was 26.70 ± 2.50 years, and the actual marital age was 26.67 ± 4.55 years. The desired and actual numbers of children were 2.55 ± 0.69 (male vs female, 1.24 ± 0.50 vs 1.38 ± 0.53) and 1.77 ± 0.84, respectively. The artificial abortion rate (total number of artificial abortions/total number of the pregnancies) was 10.96%. There were no significant differences between the desired and actual marital ages; however, the mean actual number of children was significantly lower than the mean desired number of children.

The desired and actual marital ages, desired and actual number of children, number of family members, percentage of unmarried women, and artificial abortion rates in the different generations are shown in Table 1. There were no significant differences in the desired and actual marital ages across all generations, between the women in their 20s, 30s, and 40s, and between the women older than 60 years and those in their 20s, 30s, 40s, and 50s. The mean actual marital age was significantly lower in the women in their 20s than across all generations; in the women in their 50s than in those in their 30s and 40s; and in the women older than 60 years than in those in their 30s and 40s. However, there was no significant difference between the women in their 30s and those in their 40s, and between the women in their 50s and those older than 60 years.

**Table 1:** Desired and actual marital ages, desired and actual numbers of children, number of family members, percentage of unmarried women, and artificial abortion rate stratified according to age group

	**20–29 years**	**30–39 years**	**40–49 years**	**50–59 years**	**Older than 60 years**
	**(n = 484)**	**(n = 629)**	**(n = 309)**	**(n = 109)**	**(n = 71)**
Desired marital age (years)	26.07 ± 2.35*^#†^	27.1 ± 2.37*^#†^	27.2 ± 2.53*^#†^	26.63 ± 2.48*^†^	25.59 ± 2.59*^†^
Actual marital age (years)	24.04 ± 2.75^*^	27.97 ± 3.91*^#†^	27.96 ± 5.41*^#†^	24.89 ± 4.35^#^	25.14 ± 5.07^†^
Desired number of children	2.46 ± 0.74^#^	2.53 ± 0.64^†^	2.65 ± 0.67*^#†^	2.74 ± 0.74*^#†^	2.80 ± 0.62*^#†^
(male:female)	(1.25:1.26)	(1.20:1.41)	(1.32:1.42)	(1.29:1.55)	(1.24:1.68)
Actual number of children	1.33 ± 0.67*^#†^	1.57 ± 0.81*^#†^	2.00 ± 0.96*	2.13 ± 0.68^#^	2.15 ± 0.73^†^
Number of family members	3.22 ± 1.22	3.16 ± 1.12^†^	3.64 ± 1.31*^#^	3.19 ± 1.13^#^	2.95 ± 1.26*
Unmarried women (%)	41.61	14.76	16.50	11.65	12.50
Abortion rate (%)	20.17*	8.44*^#^	7.47*^#^	9.84*^#^	16.39^#^

The data are expressed as mean ± SD values, unless otherwise indicated. The superscripted symbols (*^#†^) indicate a pair of significant difference (p < 0.05).

The mean desired number of children was significantly higher than the mean actual number of children across all generations. The mean desired numbers of children in the women in their 20s and 30s were significantly lower than those in the women in their 40s, those in their 50s, and in those older than 60 years. There was no significant difference in the desired number of children between the women in their 40s, those in their 50s, and those older than 60 years. The mean actual numbers of children of the women in their 40s, those in their 50s, and those older than 60 years were significantly greater than those of the women in their 20s and 30s. The women in their 50s and those older than 60 years had a significantly high preference for a female child. The mean actual number of children of the women in their 30s was significantly higher than that of the women in their 20s; however, there was no significant difference between the women in their 40s and 50s and those older than 60 years.

The number of family members was significantly lower for women older than 60 years than for women in their 40s. This number was lower for women in their 50s than for those in their 40s.

The mean artificial abortion rate was significantly higher among women in their 20s than among those in their 30s, 40s, and 50s; the value was also higher in the women older than 60 years than in those in their 30s, 40s, and 50s. There was no significant difference in artificial abortion rates among women in their 30s, 40s, and 50s.

**Figure 1:**
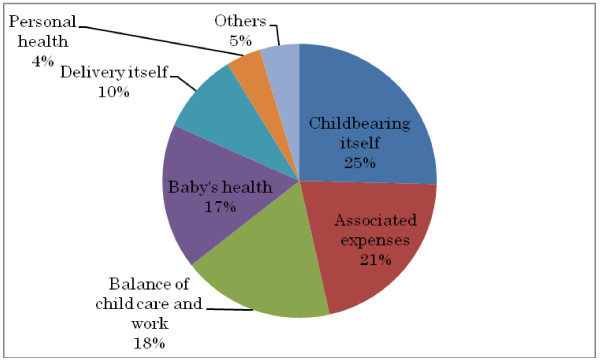
Causes of anxiety regarding pregnancy and childbearing among the women who had never delivered a baby.

The anxiety about pregnancy and childbearing among the women who had never delivered a baby is shown in Figure 1. The primary concern was regarding childbearing itself (such as whom to ask questions when in trouble), followed by finance-related concerns. Those who worried about finances were significantly younger than those who worried about their own and their baby’s health, and the balance between child care and career. In addition, those who worried about their own health were significantly older than those who worried about the health of their baby and the delivery itself (such as labor pain). Furthermore, those who worried about childbearing itself were significantly younger than those who worried about the balance between child care and career.

The age, desired marital age, desired and actual numbers of children, number of family members, and artificial abortion rate are presented in Table 2 according to marital status.

**Table 2:** Age, desired and actual marital ages, desired and actual numbers of children, number of family members, and artificial abortion rate stratified according to marital status

	**Married**	**Unmarried**
	**(n = 1234)**	**(n = 362)**
Age (years)	37.59 ± 10.83*	31.44 ± 9.83*
Desired marital age (years)	26.50 ± 2.30*	27.60 ± 2.87*
Actual marital age (years)	26.89 ± 4.49	
Desired children number (male:female)	2.59 ± 0.70*	2.43 ± 0.65*
	(1.25:1.41)	(1.20:1.31)
Actual number of children	1.74 ± 0.86	1.82 ± 0.96
Number of family members	3.25 ± 1.23*	3.49 ± 1.10*
Abortion rate (%)	8.63*	34.48*

The data are expressed as mean ± SD values, unless otherwise indicated. The asterisk indicates a pair of significant difference (p < 0.05).

The mean age of the married women was significantly older than that of the unmarried women. The desired marital age was significantly high among the unmarried women; however, the desired number of children was small. The number of family members and artificial abortion rate were significantly high for the unmarried women.

The age, desired and actual marital ages, desired and actual number of children, number of family members, percentage of unmarried women, and artificial abortion rate are presented in Table 3 according to residential area. Furthermore, the data for the women aged 30–39 and 40–49 years who were living in an urban/semi-urban/rural area were extracted for analysis and matched for age (Table 4).

**Table 3:** Age, desired and actual marital ages, desired and actual numbers of children, number of family members, percentage of unmarried women, and artificial abortion rate stratified according to residential area

	**Urban**	**Semi-urban**	**Rural**	**Island**
	**(n = 728)**	**(n = 438)**	**(n = 233)**	**(n = 57)**
Age (years)	35.77 ± 10.12*	35.49 ± 10.55*	40.05 ± 13.44*	36.02 ± 10.93*
Desired marital age (years)	27.03 ± 2.69*	26.53 ± 2.54*	26.10 ± 2.30*	26.12 ± 2.15*
Actual marital age (years)	26.75 ± 4.51*^#^	26.75 ± 4.51*^#^	25.83 ± 4.58*	25.76 ± 3.55^#^
Desired children number (male-female)	2.54 ± 0.66*	2.49 ± 0.72*^#^	2.73 ± 0.73*	2.66 ± 0.58^#^
	(1.25:1.39)	(1.21:1.33)	(1.31:1.47)	(1.24:1.50)
Actual number of children	1.70 ± 0.90*	1.60 ± 0.81*^#^	2.09 ± 0.82*^#^	2.03 ± 0.86*^#^
Number of family members	3.25 ± 1.14*^#^	3.05 ± 1.06*	3.84 ± 1.52*^#^	3.67 ± 1.16*^#^
Unmarried women (%)	28.71	16.89	16.31	31.58
Abortion rate (%)	12.68*	11.17*	6.79*	10.42

The data are expressed as mean ± SD values, unless otherwise indicated. The superscripted symbols (*^#^) indicate a pair of significant difference (p < 0.05).

**Table 4:** Age, desired and actual marital ages, desired and actual numbers of children, number of family members, and artificial abortion rate stratified according to the residential area of the women aged 30–39 and 40–49 years

**Age range, 30–39 years>**			
	**Urban**	**Semi-urban**	**Rural**
	**(n = 301)**	**(n = 182)**	**(n = 63)**
Age (years)	34.30 ± 2.72	34.03 ± 2.82	34.63 ± 2.64
Desired marital age (years)	26.77 ± 2.48	27.10 ± 2.22	26.51 ± 2.53
Actual marital age (years)	27.24 ± 4.95*	28.37 ± 4.22*^#^	27.24 ± 3.84^#^
Desired children number (male:female)	2.53 ± 0.70	2.46 ± 0.60*	2.71 ± 0.60*
	(1.27:1.38)	(1.16:1.33)	(1.23:1.51)
Actual number of children	1.66 ± 0.80	1.54 ± 0.78*	1.81 ± 0.80*
Number of family members	3.16 ± 1.10*	3.01 ± 0.91^#^	3.91 ± 1.69*^#^
Abortion rate (%)	13.87^*^	9.06	5.83*
**<Age range, 40–49 years>**			
	**Urban**	**Semi-urban**	**Rural**
	**(n = 149)**	**(n = 72)**	**(n = 57)**
Age (years)	43.93 ± 3.03	43.99 ± 3.01	44.04 ± 2.66
Desired marital age (years)	27.21 ± 2.31*	27.10 ± 2.76	26.45 ± 2.02*
Actual marital age (years)	26.30 ± 4.00*^#^	27.92 ± 5.18*	27.85 ± 6.16^#^
Desired children number (male:female)	2.54 ± 0.62*	2.56 ± 0.66^#^	2.82 ± 0.66*^#^
	(1.18:1.44)	(1.25:1.41)	(1.40:1.52)
Actual number of children	1.51 ± 0.83*^#^	1.82 ± 0.90*^†^	2.29 ± 0.79*^#†^
Number of family members	3.12 ± 0.98^*^	3.41 ± 1.17^#^	4.27 ± 1.37*^#^
Abortion rate (%)	11.76*^#^	4.84*	4.48^#^

The mean age was significantly higher among the participants from the rural area than among those from the other 3 areas. The mean desired marital age was significantly higher among the participants from the urban area than among those in the other 3 areas, among those from the semi-urban area than among those from the rural area, and among those from the island than among those from the rural area. The actual marital ages were not different between the women from the urban area and those from the semi-urban area. The mean actual marital age was significantly lower among the women from the rural area and those from the island than among those from the urban and semi-urban areas.

The desired and actual numbers of children were significantly greater for the women in the rural areas than for those in the urban and semi-urban areas. The mean desired number of children for the women in the island was greater than that for those in the semi-rural area. In addition, the mean actual number of children was greater for the women in the island area than for those in the urban and semi-urban areas. There was a significantly high preference for a female child among women from the rural and island areas.

The mean number of family members was significantly lower in the semi-urban area than in the other 3 areas; furthermore, the mean number of family members was lower in the urban area than in the rural and island areas.

The mean artificial abortion rate was significantly lower in the rural area than in the other 3 areas.

Among the women aged 30–39 years, the desired marital ages did not significantly differ in terms of residential area; however, the mean actual marital age was significantly higher for the women in the semi-urban area than for those in urban and rural areas. The desired and actual numbers of children were significantly higher for the women in the rural area than those in the semi-urban area. Among the women aged 40–49 years, the mean desired marital age was significantly younger for those in the rural area than for those in the urban area; however, the mean actual marital age was younger for the women in the urban area than for those in the semi-urban and rural areas. In addition, the mean desired number of children was significantly higher for the women in the rural area than for those in the urban and semi-urban areas. Among the residential areas, the urban area was associated with the smallest mean actual number of children, whereas the rural area had the highest number.

For the women aged 30–39 and 40–49 years, the mean number of family members is greater in rural area than in the urban and semi-urban areas and the mean abortion rate was highest in the urban area.

## Discussion

In 2010, the mean marital ages of women in Japan and in Hyogo were 28.8 and 28.7 years, respectively [4]. The mean marital age of women was 23.3 years in 1952 and has been increasing gradually thereafter [4]. Our study targeted all generations, and the mean age of our study participants was 39.96 years; therefore, the actual marital age was below the current mean marital age. Our results showed no significant difference between the mean desired (26.70 years) and actual marital ages (26.67 years); however, the mean desired marital age was significantly younger among the women older than 60 years than among those in their 20s, 30s, 40s, and 50s, indicating that the older women were more sensitive about marital age.

In high-fertility countries, the stated fertility intentions tend to be lower than the actual values; however, low-fertility countries showed just the opposite tendency [6]. In 1982, American women aged 15–44 years expected to have a mean of 2.38 children, whereas in 1988, they expected to have a mean of 2.22 children. Both of these estimates are significantly higher than the actual TFRs for the corresponding years (1.83 births in 1982 and 1.93 in 1988) [2]. Our results indicated a significant lower mean actual number of children (1.77) than the mean desired number of children (2.55). Furthermore, in comparison, the younger generation desired fewer children. This present condition in which the younger generation desires fewer children is the reason underlying Japan’s low birth rate. The same trend was observed in low-fertility European countries, suggesting that poor economic conditions, especially among the younger generation, may lower the desire to have a large family, thus making the younger population more pessimistic about their ability to find a partner and to afford having children [6]. Under the recent economic depression in Japan, the younger generation has neither the opportunity to acquire sufficient income nor the volition to bear and raise a child.

In our study, the desired and actual numbers of children were significantly greater in the rural areas than in the urban and semi-urban areas. Our questionnaire did not include questions about the educational background and economic condition of the participants; therefore, the gap between the urban and rural population with respect to education and economic conditions is unknown. Furthermore, the influence of age difference between the urban and rural residents cannot be denied; however, a gap surely exists even within the same prefecture. The number of family members reported by women older than 50 years was lower, suggesting that the women older and those younger than 50 years rarely lived together. Even the trend toward a nuclear family seems to be progressing in Hyogo; the mean number of family members is significantly higher in the rural areas than in the urban/semi-urban areas, suggesting that relatively larger families live in the rural areas.

The women who had never delivered a baby indicated that their primary concern was regarding childbearing itself (such as whom to ask questions when in trouble), which might be the key reason for the low fertility rate in Japan.

The economic development and higher education of Japan have exposed women to a wider selection of job opportunities than before; however, the role of women after marriage, which includes household tasks, childbearing, and usually, care of the elderly, has not changed [7]. Pregnancy and childbearing in Japan have been traditionally supported by members of a large family, especially mothers and grandmothers [8]. Given that the gender role at home has not changed, Japanese women still rely on the cooperation of members of a large family, and more so during pregnancy and childbirth.

In many Asian countries, such as China, India, Nepal, Bangladesh, and Pakistan, the preference for a male child is obvious [9-11]. In China, a population imbalance in favor of males was a social concern and sex-selective abortions became a major problem after the 1-child policy [12]. In Korea, the sex ratio at birth in 1970 was 109.5, which recovered to 106.2 (normal ratio, 103–107) in 2007 and 106.9 in 2010, suggesting that the preference for having a son has changed among parents [13]. It is noteworthy that in our study, male preference was not observed. Conversely, the women in their 50s and those older than 60 years and living in the rural and island areas preferred having female children.

The mean artificial abortion rate was high among the women in their 20s and those older than 60 years. It was reported that the legal induced abortion ratio (number of induced abortions per 1,000 live births) showed a gradual decline over time from 672 per 1,000 live births in 1955 to 292 between 1998 and 2001. However, the number and ratio of artificial abortion in the youngest age group (<20 years) increased from 1995 through 2001 [14].

One reason for the high artificial abortion rates among women in their 20s, as reported in a previous study, may be the inadequate or misconceptions regarding contraceptives and sexual practices among sexually active young adults. A limitation of our study is that all the participants were patients at the obstetrics and gynecology departments of clinics/hospitals; therefore, some of the participants may have visited the clinic/hospitals for the purpose of terminating pregnancy. As a result, the artificial abortion rate among the young adults in our study may be higher than that of the general population. Meanwhile, a strong point of our study is that the obstetrics and gynecology departments of clinics/hospitals provided a suitable venue where women feel comfortable to answer questions about their pregnancy and abortion history despite abortion being a very sensitive subject among young unmarried women.

Although our target population may not accurately reflect the general female population, our findings estimate the fertility intention and family size preference in Japan.

Japanese women desire more children than they actually have. To improve the fertility rate in Japan, it is important to recover the businesses and promote the employment of the younger generation. Furthermore, it is essential to create a social environment that is supportive of a conventional extended family, which young women can easily access.

Furthermore, husbands sometimes acquire parental leave. The participation of a husband in child care may be an important element that influences a working woman to consider having additional children. In Japan, pregnant mothers often stay with their parents to get assistance in delivery and child care from their own mothers (“Satogaeri”). It was reported that Japanese women were relieved of their anxiety by learning about upcoming childbearing tasks under the guidance of their own mothers [15].

Japanese women desire a healthcare system that allows for maternity and childcare leaves by other family members such as mothers of the pregnant women to support their daughters’ delivery.

## Conclusions

The family size preference in our study was higher than the actual number of children. In addition, we found that younger women preferred a small family. In our study, the fertility intentions were higher among the women living with bigger families in rural areas.

## Abbreviations

TFR: Total fertility rate.

## Competing interests

The authors declare that they have no competing interests.

## Authors’ contributions

YM made substantial contributions to the concept and design of the study and acquisition, analysis, and interpretation of data. YM was also been involved in drafting and critically revising the manuscript for important intellectual content. SY conceived and participated in the design and coordination of the study and contributed to the writing of the manuscript draft. Both authors have read and approved the final manuscript.

## References

[B1] Ding QJ Hesketh T Family size, fertility preferences, and sex ratio in China in the era of the one child family policy: results from national family planning and reproductive health survey BMJ 2006 333 7564 371 373 Epub 2006 May 11 10.1136/bmj.38775.672662.80 16690642 PMC1550484

[B2] Mosher WD Bachrach CA Understanding U.S. fertility: continuity and change in the National Survey of Family Growth, 1988-1995 Fam Plann Perspect 1996 28 1 4 12 10.2307/2135956 8822409

[B3] Fertility of Men and Women Aged 15–44 Years in the United States National survey of family growth, 2006-2010 http://www.cdc.gov/nchs/data/nhsr/nhsr051.pdf. Accessed on June13, 2012 22803225

[B4] Portal site of official statistic of Japan http://www.e-stat.go.jp/SG1/estat/eStatTopPortalE.do. Accessed on June 15, 2012

[B5] Hyogo prefecturehttp://web.pref.hyogo.jp/stat/index.html. Accessed on June 21, 2012

[B6] Goldstein JR Lutz W Testa MR The emergence of sub-replacement family size ideals in Europe Population Research and Policy Review 2003 22 479 496

[B7] Frejka T Jones GW Sardon JP East Asian childbearing patterns and policy developments Popul Dev Rev 2010 36 3 579 606 10.1111/j.1728-4457.2010.00347.x 20882707

[B8] Raymo JM Iwasawa M Bumpass L Cohabitation and family formation in Japan Demography 2009 46 4 785 803 10.1353/dem.0.0075 20084829 PMC2831360

[B9] Bhattacharjya D Sudarshan A Tuljapurkar S Shachter R Feldman M How can economic schemes curtail the increasing sex ratio at birth in China? Demogr Res 2008 19 54 1831 1850 21113272 10.4054/DemRes.2008.19.54 PMC2990196

[B10] Jayaraman A Mishra V Arnold F The relationship of family size and composition to fertility desires, contraceptive adoption and method choice in South Asia Int Perspect Sex Reprod Health 2009 35 1 29 38 10.1363/3502909 19465346

[B11] Hussain R Fikree FF Berendes HW The role of son preference in reproductive behaviour in Pakistan Bull World Health Organ 2000 78 3 379 388 10812738 PMC2560708

[B12] Zhu WX Lu L Hesketh T China’s excess males, sex selective abortion, and one child policy: analysis of data from 2005 national intercensus survey BMJ 2009 338 b1211 10.1136/bmj.b1211 19359290 PMC2667570

[B13] Lim JW The changing trends in live birth statistics in Korea, 1970 to 2010 Korean J Pediatr 2011 54 11 429 435 Epub 2011 Nov 30 10.3345/kjp.2011.54.11.429 22253639 PMC3254888

[B14] Baba S Tsujita S Morimoto K The analysis of trends in induced abortion in Japan-An increasing consequence among adolescents Environ Health Prev Med 2005 10 1 9 15 10.1265/ehpm.10.9 21432158 PMC2723632

[B15] Kobayashi Y Assistance received from parturients’ own mothers during “Satigaeri” (their perinatal visit and stay with their parents) and development of the mother-infant relationship and maternal identity J Jpn Acad Midwif 2010 24 1 28 39 10.3418/jjam.24.28

